# Determining the role of external beam radiotherapy in unresectable intrahepatic cholangiocarcinoma: a retrospective analysis of 84 patients

**DOI:** 10.1186/1471-2407-10-492

**Published:** 2010-09-14

**Authors:** Yi-Xing Chen, Zhao-Chong Zeng, Zhao-You Tang, Jia Fan, Jian Zhou, Wei Jiang, Meng-Su Zeng, Yun-Shan Tan

**Affiliations:** 1 Department of Radiation Oncology, Zhongshan Hospital, Fudan University, Shanghai, China; 2 Liver Cancer Institute, Zhongshan Hospital, Fudan University, Shanghai, China; 3 Department of Radiology, Zhongshan Hospital, Fudan University, Shanghai, China; 4 Department of Pathology, Zhongshan Hospital, Fudan University, Shanghai, China

## Abstract

**Background:**

Intrahepatic cholangiocarcinoma (ICC) is the second most common type of primary liver cancer. Only few studies have focused on palliative radiotherapy used for patients who weren't suitable for resection by surgery. This study was conducted to investigate the effect of external beam radiotherapy (EBRT) for patients with unresectable ICC.

**Methods:**

We identified 84 patients with ICC from December 1998 through December 2008 for retrospective analysis. Thirty-five of 84 patients received EBRT therapy five times a week (median dose, 50 Gy; dose range, 30-60 Gy, in fractions of 1.8-2.0 Gy daily; EBRT group); the remaining 49 patients comprised the non-EBRT group. Tumor response, jaundice relief, and survival rates were compared by Kaplan-Meier analysis. Patient records were reviewed and compared using Cox proportional hazard analysis to determine factors that affect survival time in ICC.

**Results:**

After EBRT, complete response (CR) and partial response (PR) of primary tumors were observed in 8.6% and 28.5% of patients, respectively, and CR and PR of lymph node metastases were observed in 20% and 40% of patients. In 19 patients with jaundice, complete and partial relief was observed in 36.8% and 31.6% of patients, respectively. Median survival times were 5.1 months for the non-EBRT group and 9.5 months for the EBRT group (*P *= 0.003). One-and two-year survival rates for EBRT versus non-EBRT group were 38.5% versus 16.4%, and 9.6% versus 4.9%, respectively. Multivariate analysis revealed that clinical symptoms, larger tumor size, no EBRT, multiple nodules and synchronous lymph node metastases were associated with poorer prognosis.

**Conclusions:**

EBRT as palliative care appears to improve prognosis and relieve the symptom of jaundice in patients with unresectable ICC.

## Background

Intrahepatic cholangiocarcinoma (ICC) is the second most common type of primary liver cancer. A recent study from the United States reported a 9% annual increase and an overall tenfold increase in ICC-related mortality since 1973 [1].

Lack of symptoms until late in the disease typically results in advanced ICC at the time of diagnosis, and cure rates are low for patients with advanced stage disease, even with aggressive therapy. Overall resectability rates were 54.6% in a series from Japan [2] and 62% in a series from United States [3]; 1-and 3-year cumulative survival rates in the resected cases were 49.4% to 76.6% and 17.3% to 52.7%, respectively [4-7]. Among the unresectable cases, median survival was less than 5 months [8-10], which represents a rapidly fatal process.

Surgical resection is the only potentially curative treatment; however, disease is already unresectable at presentation in some patients. Most of these patients are candidates for palliative therapy, which includes biliary drainage, systemic chemotherapy, transarterial chemoembolization (TACE), and photodynamic therapy. However, cholangiocarcinomas respond poorly to existing therapies, so these palliative options are of limited benefit [11].

Although a few studies have focused on palliative radiotherapy for these patients, no controlled trials have been conducted. The purpose of the current study was to evaluate the clinical effectiveness of radiotherapy in patients with unresectable ICC by retrospective analysis and to identify prognostic factors associated with clinical outcomes.

## Methods

During the period from December 1998 to December 2008, a total of 549 patients with ICC were hospitalized in the Liver Cancer Institute, Zhongshan Hospital, Fudan University, among which 406 patients underwent hepatectomy, and the remaining 143 were unresectable.

Unresectable ICC was determined by surgeons and the following clinical conditions were considered to be unresectable: extensive bilobular involvement of the liver by a large solitary tumor or by multiple tumors, or invasion of major blood vessels. Of 143 unresectable patients, 59 were excluded from this study, among which 14 patients had Child-Pugh C or other serious diseases, and 45 patients were diagnosed by clinic presentation. The remaining 84 patients were confirmed by histological examination combined with medical history and excluded from HCC and metastases from gastrointestinal cancers by immunohistochemical staining of CK7, CK19, CK20, AFP, MUC5AC, MUC6 and Hepa (Table 1). Abdominal and pelvic examination by CT/MRI scan or endoscopy was also performed to confirm no evidence of gastrointestinal cancers; tumor markers such as carcinoembryonic antigen (CEA), carbohydrate antigen 19-9 (CA19-9), and alphafetoprotein (AFP) were also used to differentiate ICC from other liver tumors.

**Table 1:** Immunochemical analysis of liver tumors.

	Hepa	CK7	CK19	CK20	AFP	MUC5AC	MUC6
Hepatocellular carcinoma	+	-	-	-	+/-	-	-
Hepatic metastasis from gastric cancer	-	+	+	-	-	+/-	-
Hepatic metastasis from colorectal cancer	-	-/+	-	+	-	-	+
Intrahepatic cholangiocarcinoma	-	+	+	-	-	+	-

Among 84 patients included in this study, 35 patients receiving external beam radiotherapy (EBRT; EBRT group) and 49 patients did not receive radiotherapy (control non-EBRT group). In EBRT group, one patient (2.9%) belonged to TNM-I stage, followed by 8 to TNM-II stage (22.9%), 9 to TNM-III stage (25.7%), 12 to TNM-IVa stage (34.3%), and 5 to TNM-IVb stage (14.3%), according to the 7th edition of UICC-TNM staging system for ICC [12]; In non-EBRT group, six patients (12.2%) belonged to TNM-II stage, followed by 10 to TNM-III stage (20.4%), and 29 to TNM-IVa (59.2%), and 4 to TNM-IVb stage (8.2%). Transarterial chemoembolization (TACE) was also used in 28 patients, including 15 patients from EBRT group and 13 from non-EBRT group. We identified these patients to determine the role of TACE in the treatment of unresectable ICC. Because the role of chemotherapy is undefined in ICC, no systemic chemotherapy was administered during the entire treatment period. This study was approved by the ethical review board of Zhongshan Hospital, Fudan University and in compliance with the Helsinki Declaration of 1975, as revised in 2000.

ICC is defined as a tumor arising peripheral to the secondary bifurcation of the left or right hepatic duct. Thus, tumors arising from the right and/or left hepatic ducts were excluded from the study. However, there were significant differences between the clincopathological features of ICC and outcome in patients with tumors located close to the hilarum peripherally [13]; accordingly, ICC was topographically divided into two types: a central type, in which the carcinoma was related to a major intrahepatic bile duct, and a peripheral type. Synchronous lymph node (LN) metastasis was defined as a diagnostic interval between ICC and extrahepatic LN metastasis of not longer than 1 month. Patient demographics and baseline characteristics are displayed in Table 2.

**Table 2:** Patient demographics and baseline characteristics.

	Non-EBRT (*n *= 49)	EBRT (*n *= 35)	*P*-value
Sex, *n *(%)			0.308
Male	32 (65.3%)	19 (54.3%)	
Female	17 (34.7%)	16 (45.7%)	
Age, *n *(%)			0.631
≤60 years old	25(51.0%)	16 (45.7%)	
>60 years old	24(49.0%)	19 (64.3%)	
Clinical symptoms, *n *(%)			0.386
Asymptomatic	9(18.4%)	4 (11.4%)	
Symptomatic	40(81.6%)	31 (88.6%)	
Diameter, mean ± SE (cm)	8.6 ± 3.4	7.7 ±3.2	0.238
≤5, *n *(%)	6(12.2%)	7 (20.0%)	0.280
5-10, *n *(%)	21 (44.9%)	18 (51.4%)	
≥10, *n *(%)	22 (44.9%)	10 (28.6%)	
Intrahepatic lesions, *n *(%)			0.898
Solitary	37(75.5%)	26 (74.3%)	
Multiple nodules	12 (24.5%)	9 (25.7%)	
Tumor Types, *n *(%)			0.722
Peripheral ICC	34 (69.4%)	23 (65.7%)	
Central ICC	15 (30.6%)	12 (34.3%)	
Synchronous LN metastases, *n *(%)			0.064
No	18 (36.7%)	20 (57.1%)	
Yes	31 (63.3%)	15 (42.9%)	
CA19-9, U/mL			0.676
≤37, *n *(%)	9 (18.4%)	4 (11.4%)	
37-600, *n *(%)	15 (30.6%)	11 (31.4%)	
≥600, *n *(%)	25 (51.0%)	20 (57.1%)	
TACE, *n *(%)			0.118
Yes	13(26.5%)	15(42.9%)	
No	36(73.5%)	20(57.1%)	
TNM stage, *n *(%)			0.175
I		1(2.9%)	
II	6(12.2%)	8(22.9%)	
III	10(20.4%),	9(25.7%)	
IVA	29(59.2%)	12(34.3%)	
IVB	4(8.2%)	5(14.3%)	

EBRT, external beam radiotherapy; TACE, transcatheter arterial chemoembolization; SE, standard error; ICC, intrahepatic cholangiocarcinoma; LN, lymph node.

### Therapies

**Figure 1:**
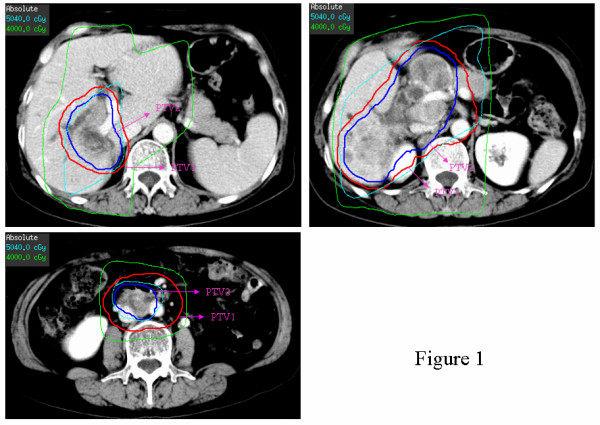
**Three-dimensional conformal radiation therapy for intrahepatic cholangiocarcinoma (ICC) patient with asynchronous hepatic portal, peri-pancreas, and para-aorta lymph node metastases, showing delineation of plan tumor target volume 1 (PTV1) and plan target volume 2 (PTV2) for 50.4 Gy/28 Fx and 41.4 Gy/23 Fx, dose distribution, and dose volume histogram (DVH) for tumor targets and organs at risk**.

Each patient provided written informed consent regarding the treatment course. Patients received limited-field EBRT using a linear accelerator with 6-or 15-MV photons. Prior to June 2005, traditional (two-dimensional) design and delivery of EBRT relied on a CT/MRI scan of the abdomen to show the position of the tumor using a simulation process (n = 13) [14]. Since July 2005, three-dimensional conformal radiation therapy was used (n = 22). For EBRT planning, the patients underwent CT in the supine position with both arms raised above the head; CT images were then transferred to a three-dimensional conformal radiation therapy planning system (Pinnacle 7.6C). Gross tumor volume (GTV) included the primary tumor and involved lymph nodes. Clinic target volume (CTV) was defined as GTV with a 5-mm margin, and included regional nodes areas for LN involvement. Planning target volume (PTV) was defined as clinical target volume with a 5-to 7-mm margin to account for daily setup error, with cephalic and caudal margin enlargement (1-1.5 cm) for respiratory motion. Figure 1 represents a typical case demonstrating targeting volume.

We scheduled the full radiation dosage up to 50 to 60 Gy. For patients with synchronous LN metastasis, typical EBRT practice in our department was to deliver 40 Gy to the PTV with 10 to 20 Gy delivered to the GTV as boost fields. A reduced dose was considered after evaluating adverse effects, liver function, and distant metastases during EBRT, and an increased dose was delivered if patients tolerated therapy well (five times a week; dose range, 30-60 Gy; daily fraction, 1.8-2.0 Gy; Table 3). If all or part of the right kidney was in the radiation field, an initial left renal evaluation with intravenous pyelography was performed to ensure adequate function of the left kidney. When the duodenum was encompassed within the EBRT fields, the total EBRT dose was limited to ≤ 54 Gy.

**Table 3:** External beam radiotherapy (EBRT) treatment.

Radiotherapy characteristics	*n *(%)
Radiation dose (Gy)	
30-48	5 (14.3%)
50	21(60.0%)
52-60	9(25.7%)
Irradiation Site	
Primary tumor only	20 (57.1%)
Primary tumor and LN metastases	15 (42.9%)
	
PTV volumes* (cm3)	
Minimum.	58.76
Maximum.	1087.65
Mean ± SD	382.29 ± 323.92

LN, lymph node; PTV, planning target volume; SD, standard error.

*The data of PTV volumes were only gathered from the patients who received three-dimensional conformal radiation therapy.

TACE is a combination of targeting chemotherapy with 5-fluorouracil (1 g), cisplatin (80 mg), mitomycin C (10 mg), and arterial embolization with10 mL iodized oil (Lipiodol Ultra Fluid; Laboratory André Guerbet, Aulnay-sous-Bois, France) mixed with 10 mg mitomycin C, which produces both a selective ischemic effect and a chemotherapeutic effect on primary liver cancer.

### Follow-up, assessment of response, and toxicity

Pretreatment evaluation included a medical history and physical examination; complete blood cell count; liver function test; AFP, CEA, or CA 19-9 (Roche Diagnostics, Indianapolis, IN); abdominal ultrasonography; and enhanced CT or MRI. Clinical monitoring was performed weekly. Patients were advised to return for an initial follow-up examination 6 weeks after EBRT completion, during which irradiation responses were evaluated by abdominal enhanced CT scan or MRI. Patients were monitored every 3 months thereafter. Complete response (CR) was defined as complete tumor disappearance based on radiographic evidence; partial response (PR) required a ≥ 50% reduction in the sum of the products of the tumor's longest diameter and its perpendicular by CT or MRI. Stable disease (SD) indicated a < 50% decrease or a < 25% increase in the product of the longest perpendicular diameters of measurable tumors. Progressive disease (PD) was defined as an increase of ≥ 25% in the sum of the products of the tumor's longest diameter and its perpendicular, compared with the lowest value recorded, or as death from ICC within 3 months. Objective response was calculated for CR and PR; no response was calculated for SD or PD.

Twenty-five patients in the EBRT-group exhibited jaundice before treatment. Complete jaundice relief was defined as disappearance of symptoms and total bilirubin at normal level (≤17.1 μmol/L), partial jaundice relief was defined as disappearance of symptoms but total bilirubin higher than normal ( > 17.1 μmol/L), but more than two-fold lower than the pre-radiation level. Seven patients in the EBRT-group who presented with jaundice before EBRT were first referred to ultrasonography for decompression via percutaneous transhepatic catheter placement and then treated with EBRT. Jaundice relieved without catheter placement after the completion of EBRT was described as "jaundice was relieved."

The overall survival period was defined as the period from the date of ICC diagnosis to the date of death or the last follow-up appointment. Toxicity was evaluated according to the Radiation Therapy Oncology Group (RTOG) Toxicity Criteria, Version 2.0 [15]. Radiation-induced liver disease(RILD)was defined as either a minimum two-fold increase in anicteric elevation of ALP and non-malignant ascites or a minimum five-fold increase in elevated transaminases over the upper limit of normal or of pretreatment levels (Grade 3 or 4 hepatic toxicity by RTOG toxicity criteria) within 4 months after completion of radiotherapy.

### Statistical analysis

Survival analyses were carried out with the Kaplan-Meier method. We considered the date of ICC diagnosis as time zero, and patients alive at the end of follow-up were considered censored. Chi-square (χ^2^) test was used to compare the prevalence of ICC characteristics between the two groups and to assess dose-response relationships. Multivariate analysis of survival was carried out with Cox's regression model, and all variables were entered in a single step using backward stepwise regression (likelihood ratio). *P *< 0.05 was considered significant. All calculations were performed using SPSS version 15.0 for Windows (Chicago, IL).

## Results

### Response to EBRT

Of the 35 patients with unresectable ICC who underwent EBRT, primary tumors were irradiated in 35 patients: three patients (8.6%) achieved a CR, and ten (28.5%) achieved a PR at the first follow-up exam, resulting in an objective response rate of 37.1%. SD was observed in 17 patients (48.6%), with an overall disease control rate of 85.7%. Of the 35 patients, 15 received TACE. Among these 15 patients, one (6.7%) achieved CR and four (26.7%) showed PR, with an overall objective response rate of 33.3%. SD was observed in 8 patients (53.3%), with an overall disease control rate of 86.7%.

LN metastases were irradiated in 15 patients: three patients (20%) achieved a CR, and six (40%) achieved a PR, resulting in an objective regression rate of 60%.

Of the 19 patients in the EBRT-group with jaundice symptom before EBRT, jaundice was completely relieved in seven patients (36.8%) and partially relieved in six patients (31.6%) after EBRT completion. The total relief rate was 68.4%.

### Overall survival analysis and prognosis factors

At the time of analysis, 78 patients had died, 1 was lost to follow-up, and 5 were still alive. The median survival time for all patients was 6.8 ± 0.9 months.

**Figure 2:**
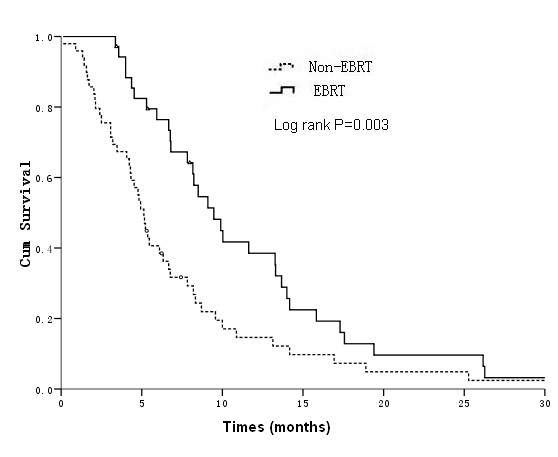
**Overall survival curves for EBRT and non-EBRT groups**.

In this study, 1-and 2-year survival rates for patients with unresectable ICC treated with EBRT (*n *= 35) compared with the non-EBRT group (*n *= 49) were 38.5% versus 16.4%, and 9.6% versus 4.9%, respectively. Median survival times were 9.5 ± 1.1months versus 5.1 ± 0.3 months, respectively (log-rank *P *= 0.003) (Figure 2).

One-and two-year survival rates for patients with unresectable ICC treated with TACE (*n *= 28) compared with those who did not undergo TACE (*n *= 56) were 39.9% versus 16.6%, and 4.0% versus 8.3%, respectively. Median survival times were 10.0 ± 1.4 months versus 5.9 ± 0.7 months, respectively (log-rank *P *= 0.201). Furthermore, In non-EBRT group, survival rates at 1 and 2 years were 18.5%/13.4%, and 0%/6.7% for treatment with TACE (n = 13) and non-TACE (n = 36), respectively; Median survival times were 5.4 ± 3.1 months versus 4.9 ± 0.3 months, respectively (log-rank P = 0.867). In EBRT group, survival rates at 1 and 2 years were 57.8%/22.7%, and 7.2%/11.4% for treatment with TACE (n = 15) and non-TACE (n = 20), respectively; Median survival times were 13.3 ± 1.5 months versus 7.8 ± 1.0 months, respectively (log-rank P = 0.268).

For the patients who did not undergo EBRT, median survival times for peripheral ICC (*n *= 34) and central ICC (*n *= 15) were 5.4 ± 0.7 months and 3.5 ± 1.1 months, respectively (*P *= 0.103). In contrast, median survival times for peripheral ICC (*n *= 23) and central ICC (*n *= 12) in the EBRT group were 8.5 ± 0.9 months and 13.3 ± 3.6 months, respectively (*P *= 0.205), and the tumor response rates were 26.1% (*n *= 6) and 58.3% (*n *= 7), respectively (*P *= 0.061). In addition, the mean tumor diameter in central ICC was significantly smaller compared with that of peripheral ICC (9.0 ± 3.4 cm versus 6.7 ± 2.7 cm (*P *= 0.003) (Table 4).

**Table 4:** Survival time and response rate in central and peripheral intrahepatic cholangiocarcinoma (ICC).

	Central ICC	Peripheral ICC	*P*-value
Median survival time (months)			
Non-EBRT group	3.5 ± 1.1	5.4 ± 0.7	0.103
EBRT group	13.3 ± 3.6	8.5 ± 0.9	0.205
CR+PR, *n *(%)	7 (58.3%)	6 (26.1%)	0.061
Diameter of tumor (cm)	6.7 ± 2.7	9.0 ± 3.4	0.003
Non-EBRT group	7.3 ± 2.5	9.2 ± 3.6	0.071
EBRT group	5.9 ± 2.9	8.7 ± 3.1	0.015

ICC, intrahepatic cholangiocarcinoma; EBRT, external beam radiotherapy; CR, complete response; PR, partial response.

The results of multivariate survival analysis are displayed in Table 5. Five factors appear to be independently associated with poorer prognosis: clinical symptoms, synchronous LN metastases, larger tumor size, multiple nodules and no EBRT. However, TACE was not an independent factor for unresectable ICC (RR = 0.893, P = 0.689).

**Table 5:** Univariate and multivariate survival analysis.

Variables	*n*	Survival Status			Univariate	Multivariate	
		**1-Year** **(*n*)**	**2-Year** **(*n*)**	**Median survival (months)**	***P***	**RR**	***P***

Sex							
Male	51	24.2	4.4	7.8 ± 1.6		1	
Female	33	25.6	11.0	6.7 ± 0.5	0.862	1.054	0.845
Age							
≤60 years old	41	33.1	5.5	7.8 ± 1.6		1	
>60 years old	43	16.5	8.2	6.7 ± 0.8	0.844	1.451	0.153
Clinical symptoms							
Asymptomatic	15	21.8	6.7	6.7 ± 0.5		1	
Symptomatic	69	66.7	19	13.1 ± 1.1	0.001	3.923	< 0.001
Diameter (cm)							
≤5	13	53.8	23.1	13.3 ± 2.8		1	
5-10	39	15.0	3.0	6.8 ± 0.5		2.476	0.025
≥10	32	23.6	3.9	5.2 ± 0.4	0.013	3.165	0.011
Intrahepatic lesions							
Solitary	63	26.6	7.1	6.8 ± 1.0		1	
Multiple nodules	21	18.5	6.2	5.2 ± 0.7	0.604	1.794	0.050
Tumor types							
Peripheral ICC	57	20.8	6.2	6.7 ± 1.0		1	
Central ICC	27	31.9	8.0	6.8 ± 2.5	0.843	1.663	0.079
LN metastases							
Asynchronous	13	69.2	15.4	14.2 ± 2.3		1	
Synchronous	71	15.5	5.2	5.9 ± 0.7	0.002	1.794	0.025
CA19-9 (U/L)							
≤37	13	36.9	18.5	7.8 ± 2.4		1	
37-600	26	32.6	4.1	9.6 ± 1.4		1.639	0.232
≥600	45	16.4	5.5	5.5 ± 0.7	0.307	1.343	0.451
EBRT							
Yes	35	38.5	9.6	9.5 ± 1.1		1	
No	49	14.6	4.9	5.1 ± 0.3	0.003	2.962	< 0.001
TACE							
Yes	28	39.9	4.0	10.0 ± 1.4		1	
No	56	16.6	8.3	5.9 ± 0.7	0.201	0.893	0.689

RR, relative risk; ICC, intrahepatic cholangiocarcinoma; LN, lymph node; EBRT, external beam radiotherapy; TACE, transarterial chemoembolization.

### Failure pattern

Thirty-two of 35 patients in the EBRT group died; causes of death included liver failure induced by tumor progression (*n *= 22, 68.8%), liver failure induced by radiation-induced liver failure (*n *= 1, 3.1%), distant metastases (lungs or brain) (*n *= 5, 15.6%), abdominal LN metastases (*n *= 2, 6.3%), cancer cachexia (*n *= 2, 6.3%).

Forty-six of 49 patients in the non-EBRT group died; causes of death included liver failure induced by intrahepatic tumor progression (*n *= 35, 76.1%), distant metastases (lungs or brain) (*n *= 4, 8.7%), abdominal LN metastases (*n *= 5, 10.9%), cancer cachexia (*n *= 1, 2.2%), and unknown cause (*n *= 1, 2.2%). There was no significant difference in main cause of death between these two groups (P = 0.344).

### Adverse events

Table 6 lists adverse events during and after EBRT. Adverse events from EBRT included increased liver enzymes after the completion of EBRT, but most of them were less than grade 2. Hematologic adverse events were not severe; some may have been due to portal hypertension and associated hypersplenism, and the lower hemoglobin may have resulted from malnutrition. Nine patients (9/13) had nausea/vomiting during the last course of EBRT. One patient developed RILD and eventually developed liver failure resulting in mortality.

**Table 6:** Adverse events in the patients receiving external beam radiotherapy (EBRT group, *n *= 35).

	RTOG toxicity grade			
	
Adverse events	1 *n *(%)	2 *n *(%)	3 *n *(%)	4 *n *(%)
Gastrointestinal				
Anorexia	9 (25.7%)	3 (8.6%)	1 (2.9%)	
Nausea	10 (28.6%)	3 (8.6%)		
Vomiting	4 (11.4%)	2 (5.7%)		
Hepatic				
ALT	5 (14.3%)	2 (5.7%)	1 (2.9%)	
AST	4 (11.4%)	1 (2.9%)		
ALP	5 (14.3%)	2 (5.7%)	1 (2.9%)	
Bilirubin	3 (8.6%)	1 (2.9%)		
Bone Marrow				
WBC	4 (11.4%)	2 (5.7%)		
RBC	2 (5.7%)	1 (2.9%)		
PLT	4 (11.4%)	3 (8.6%)	1 (2.9%)	

ALT, alanine aminotransferase; AST, aspartate aminotransferase; ALP, alkaline phosphatase; RTOG, Radiation Therapy Oncology Group; WBC, white blood cell; RBC, red blood cell; PLT, platelets.

## Discussion

Results of the present study demonstrate that EBRT was associated with improving survival in patients with unresectable ICC. Patients with central ICC appear to benefit more from radiation compared with patients with peripheral ICC.

Many authors have concluded that an aggressive surgical approach is warranted in patients with peripheral ICC because resection offers the only hope for long-term survival [4-7,16]. Unfortunately, most patients present with advanced disease because peripheral ICC is usually asymptomatic in an earlier stage. Recently, the majority of studies that showed benefits from therapy used a combination of EBRT and intraluminal iridium (Ir^192^) for patients with extrahepatic cholangiocarcinoma [17]. However, few reports have focused on intrahepatic cholangiocarcinoma, and no controlled trials have evaluated the combination of EBRT and intraluminal iridium in that patient group. This is partially because transcatheter brachytherapy boosts are not feasible in the patients with peripheral or intrahepatic hilar cholangiocarcinoma; other reports show no survival benefit for EBRT in ICC [18].

Experience with EBRT is growing, but few reports provide data needed to compare treatment with and without EBRT in patients with unresectable ICC. Therefore, radiotherapy in unresectable ICC was evaluated in this 10-year retrospective study. The findings of the present study are useful not only for assessing patient prognosis but also for evaluating the efficacy of palliative treatments. EBRT can be administered for local tumor control in an attempt to improve the survival time while maintaining an acceptable quality of life.

Among patients received radiotherapy, median survival time associated with central ICC was longer compared with peripheral ICC (13.3 versus. 8.5 months). A possible reason is that central ICC is closer to the hilus hepatis and is prone to obstructing the common bile duct, causing jaundice and leading to an earlier diagnosis. In contrast, lack of special symptoms such as jaundice or pain in the earlier stages may lead to larger tumors in peripheral ICC at diagnosis. In the present study, mean tumor diameters in central and peripheral ICC were 6.7 ± 2.7 cm and 9.0 ± 3.4 cm (*P *= 0.003), respectively. Because a higher control rate is typically obtained for smaller tumors using the same radiation dose, response to radiotherapy (CR+PR) for central ICC was better than peripheral ICC (central ICC, 58.3%; peripheral ICC, 26.1%; *P *= 0.061).

Systemic chemotherapy for cholangiocarcinoma is administered to patients who are not candidates for surgery, gemcitabine or fluoropyrimidine are commonly utilized for the treatment of advanced disease. Although chemotherapy has been reported to be more beneficial than the best supportive care [19], randomized, prospective trial data were lacking in this disease, no standard chemotherapy regimen has yet been established [20]. However, data regarding chemotherapy are disappointing, but new regimen or combination of targeted therapy has been showed a promising result recently [21-23]. Systemic chemotherapy can be used for metastatic disease, and chemotherapy combined with EBRT may have an important role in preventing distant metastases and enhancing the radiation effects. Unfortunately, since its disappointing efficacy before, chemotherapy was not recommended after the diagnosis of ICC for patients in this study. This is a limitation for our retrospective analysis, and also the direction we will study next.

TACE has become an acceptable palliative treatment for patients with unresectable HCC. Compared with systemic chemotherapy, TACE reduces systemic side effects by increasing the local concentration of chemotherapeutic agents to specifically target cancer cells without damaging healthy liver tissue. However, cholangiocarcinoma is generally considered a hypovascular tumor; therefore, the efficacy of TACE for treating cholangiocarcinoma is questionable, because chemotherapeutic agents or embolic materials may not be much more effectively delivered to the tumor. In the present study, the median survival for ICC patients receiving TACE was 10.0 months, compared with 5.9 months for patients not receiving TACE (log-rank *P *= 0.201), multivariate survival analysis revealed that TACE treatment was not an independent factor affecting survival (*P *= 0.893). In EBRT group, Median survival times for treatment with TACE and non-TACE were 13.3 ± 1.5 months versus 7.8 ± 1.0 months, which might suggest that EBRT combined with TACE could improve the over all survival time of unresectable ICC, though there was no significant difference between these two groups (log-rank P = 0.268). Recently, some authors have reported improved survival for selected patients with unresectable hypervascular ICC treated with TACE [24,25]. Thus, further study is needed to determine which types of ICC are suitable for TACE therapy.

Radiation has historically played only a minor role in the management of patients with unresectable liver cancer, primarily because of low tolerance of the whole liver to irradiation. In addition to liver toxicities, normal tissues adjacent to the liver, including the stomach, duodenum, and kidneys are at risk for radiation injury if these organs can not be spared from the dose. The design of radiation fields with CT scanning has permitted the delivery of a higher radiation dose (50-60 Gy/25-30 fractions) to localized tumors. Data from HCCs during the same period in our institute reveal an overall response rate of 76% for confined intrahepatic HCC [26] and 96.8% for LN metastases from HCC [27]. HCC is more sensitive to radiation than ICC. In the present study, the main failure pattern is still local recurrence or intrahepatic metastases (68.8% versus 76.1%) with additional EBRT or without, thus new EBRT techniques or concurrence of chemotherapy are needed in the future.

In our study, we used conventional fractionated RT (1.8-2.0 Gy per fraction, five fractions a week), because liver was considered a late responding normal organ with a low ratio of α/β, which means hypofractionated RT may increase the later toxicity, but in China, hypofractionated RT has been quite often employed. In a recently published study, Liang et al [28] reported 128 cases of primary liver cancer treated by hypofractionated 3DCRT (4-5 Gy per fraction, three fractions a week) with a median total dose of 53.6 Gy. However, 15% RILD occurred (19/128), which could be attributed to large fraction size and high total RT dose. On the other hand, the development of stereotactic body radiotherapy (SBRT) has substantially improved the spatial distribution of the administered dose (tumor dose/healthy tissue dose ratio > 1). They have also opened up the immediate possibility of using higher doses per fraction than in conventional regimens, because there is less need to protect late-responding tissues if the dose they absorb can be reduced, some reports have showed safety and efficacy for intra-hepatic tumors [29,30].

Several limitations exist in the present study. First, this evaluation was carried out retrospectively, which implies all the disadvantages associated with retrospective studies. Thus, a prospective randomized trial is needed to confirm our results. Second, the appropriate rational dose for ICC is still unknown. In the present study, we used 50-60 Gy as the standard dose, but the actual dose varied from 30 Gy to 60 Gy. Therefore, caution must be used when interpreting our findings, and bias due to a positive natural course present in an individual patient has to be taken into consideration.

## Conclusions

In conclusion, due to the lack of other effective therapies, radiotherapy could be used to treat unresectable ICC to relieve symptoms such as jaundice and improve survival. Patients with central ICC appear to benefit more from radiation compared with patients with peripheral ICC.

## Competing interests

The authors declare that they have no competing interests.

## Authors' contributions

ZCZ organized the study, planned the experiments, performed the statistical analysis and helped to write the manuscript. YXC contributed to the design of the study, performed the statistical analysis, and drafted the manuscript. ZYT, JF and JZ participated in the design and coordination of the study. WJ selected the samples. MSZ contributed to analysis of the radiological data. YST contributed to analysis of the pathological data. All authors read and approved the final manuscript.

## Pre-publication history

The pre-publication history for this paper can be accessed here:

http://www.biomedcentral.com/1471-2407/10/492/prepub
